# Hip anteroposterior radiographic indices for effective osteoporosis screening in female patients undergoing total hip arthroplasty

**DOI:** 10.1007/s11657-026-01760-3

**Published:** 2026-08-28

**Authors:** Ryo Higuchi, Keisuke Uemura, Tomoki Asano, Sotaro Kono, Hirokazu Mae, Tetsuro Tani, Kazuma Takashima, Ryota Nakaya, Yasunori Okamoto, Katsuya Nakata, Seiji Okada, Hidetoshi Hamada

**Affiliations:** 1 Department of Orthopaedic Surgery, Osaka University Graduate School of Medicine, 2-2 Yamadaoka, Suita, Osaka 565-0871 Japan; 2 Department of Orthopaedic Medical Engineering, Osaka University Graduate School of Medicine, 2-2 Yamadaoka, Suita, Osaka 565-0871 Japan; 3 Department of Orthopaedic Surgery, Japan Community Health Care Organization Osaka Hospital, 4-2-78 Fukushima, Fukushima-ku, Osaka, Osaka 553-0003 Japan

**Keywords:** Total hip arthroplasty, Radiographic indices, Osteoporosis, Screening, Bone mineral density

## Abstract

***Summary*:**

Osteoporosis in patients awaiting total hip arthroplasty remains undiagnosed. Although hip radiographic indices have been proposed for screening, they have not been compared. We compared the diagnostic accuracy of eight indices for osteoporosis. The canal–bone ratio measured 7 cm below the lesser trochanter may be a useful screening tool.

**Purpose:**

Osteoporosis increases the risk of perioperative and postoperative fractures in patients undergoing total hip arthroplasty (THA). However, osteoporosis remains undiagnosed and untreated preoperatively. Several indices measured on hip anteroposterior radiographs have been proposed to screen for osteoporosis. Nevertheless, few studies have compared these indices, necessitating investigations to identify a practical and effective radiographic index.

**Methods:**

Osteoporosis prevalence was assessed in 317 female patients who underwent dual-energy X-ray absorptiometry before primary THA. The screening performance of eight radiographic indices was evaluated using anteroposterior hip radiographs for patients without previous diagnosis or treatment of osteoporosis: canal flare index (CFI), morphological cortical index (MCI), canal–bone ratio (CBR) at 2, 7, and 10 cm below the lesser trochanter and canal–bone area ratio (CBAR) measured at 2–7, 2–10, and 7–10 cm segments. Receiver operating characteristic analyses were performed, and the areas under the curve (AUCs) were calculated and compared among indices.

**Results:**

Osteoporosis was identified in 129 patients (40.7%), of whom only 48 (37.2%) received treatment. The highest AUC values were observed for CBAR2–7 (0.801), CBAR7–10 (0.798), and CBR7 (0.798), followed by CBAR2–10 (0.785), CBR10 (0.766), CBR2 (0.740), CFI (0.696), and MCI (0.654). CBAR and CBR7 exhibited higher screening accuracy than other indices.

**Conclusion:**

The osteoporosis prevalence and treatment rates were 40.7% and 37.2%, respectively. Among the radiographic indices, CBR7 and CBARs demonstrated favorable screening performance, with CBR7 offering comparable accuracy and greater simplicity. CBR7 may serve as a practical and efficient screening tool for osteoporosis.

**Supplementary Information:**

The online version contains supplementary material available at https://doi.org/10.1007/s11657-026-01760-3.

## Introduction

With the aging population, osteoporosis prevalence is increasing globally, and osteoporosis is estimated to affect 15.9 million individuals in Japan [1–3]. Osteoporosis increases the risk of perioperative and postoperative complications in patients undergoing total hip arthroplasty (THA). Therefore, adequate diagnosis and treatment before and after surgery are crucial to avoid fractures and achieve favorable outcomes [4–6]. The osteoporosis prevalence in patients undergoing THA reportedly varies depending on patient characteristics (e.g., older age and low body weight) and ranges from 8 to 25% [7–10]. Approximately 63%–78% of these patients remain undiagnosed or untreated, highlighting the importance of clarifying the osteoporosis prevalence in each patient cohort and establishing an effective method to screen for osteoporosis.

Osteoporosis is usually diagnosed by measuring the bone mineral density (BMD) using dual-energy X-ray absorptiometry (DXA) [11, 12]. However, not all patients undergoing THA receive DXA examination because of its cost and limited accessibility. Thus, several studies have employed anteroposterior hip radiographs to screen for osteoporosis. For example, researchers have used the classification proposed by Singh et al. and Dorr et al. [13, 14]. However, these indices have shown limited accuracy in predicting osteoporosis, which may result from these indices being defined descriptively and subjectively. Therefore, recent studies have used several objective morphometric indices to predict osteoporosis, including the canal–calcar ratio [15], canal flare index (CFI) [16], morphological cortical index (MCI) [17], cortical thickness index (CTI)/canal–bone ratio (CBR) [18, 19], canal–bone area ratio (CBAR) [20], and canal–diaphysis ratio [21]. Although each index has shown its ability to screen for osteoporosis [20–22], there remains a paucity of research with regard to the optimal level at which to measure these indices (e.g., 7 or 10 cm below the lesser trochanter) and which parameter is superior when several indices are compared.

To this end, the present study aimed to (1) determine the prevalence of osteopenia and osteoporosis in patients undergoing THA and investigate the preoperative treatment rate of osteoporosis, and to (2) evaluate and compare the performance of radiographic indices at various femoral levels to screen for osteoporosis.

## Materials and methods

### Analysis of osteopenia and osteoporosis prevalence and osteoporosis treatment rate

Considering that osteoporosis is a disease that is predominantly found in females, a total of 434 consecutive female patients who underwent primary THA at our institution between January 2018 and July 2024 were initially included. We excluded 117 patients who had previous hip surgery (47 patients), fractures (three patients), insufficient data (12 patients), history of corticosteroid use (33 patients), or rheumatoid arthritis (22 patients). Thus, the study cohort included 317 patients undergoing THA who were investigated for the prevalence of osteopenia and osteoporosis using DXA of the hip; osteoporosis treatment rates were also assessed according to the resulting diagnoses.

For these patients, DXA (Discovery A, Hologic Japan, Tokyo, Japan) was used to preoperatively measure the BMD of the proximal femur on the surgical side to assist the surgeon in selecting an appropriate surgical implant. The lower T-score of the femoral neck or total region was adopted to classify the analyzed femur as osteopenia or osteoporosis (osteopenia: − 2.5 < T-score ≤  − 1; osteoporosis: T-score ≤  − 2.5), following the guidelines of the International Society for Clinical Densitometry [23] with modifications for Japanese [12]. In addition, patients receiving osteoporosis treatment were classified as having osteoporosis regardless of their current T-score. In the analysis of osteoporosis treatment rate, patients who were not receiving osteoporosis treatment were defined as “untreated,” those who were receiving treatment but had a T-score ≤  − 2.5 were defined as “undertreated,” and those who were receiving treatment but had a T-score of >  − 2.5 were defined as “adequately treated.”

### Analysis for determining the osteoporosis screening accuracy using radiographic indices

In this section, the ability of radiographic indices to screen for osteoporosis was evaluated. A total of 48 patients who received osteoporosis treatment were excluded, leaving 269 female patients (269 hips) to be analyzed.

**Fig. 1:**
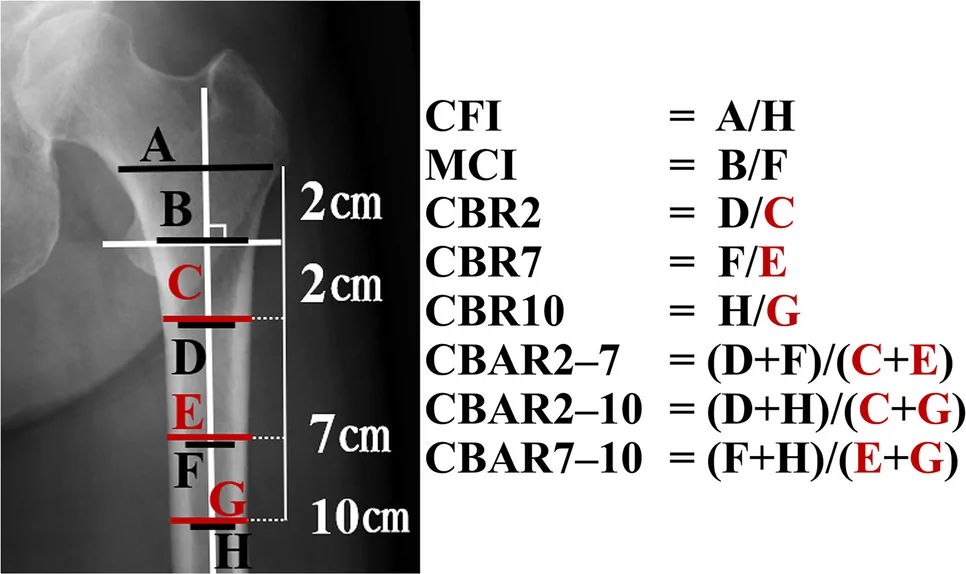
Indices measured on anteroposterior hip radiograph, including the canal flare index (CFI); morphological cortical index (MCI); canal–bone ratios at 2 (CBR2), 7 (CBR7), and 10 cm (CBR10) below the lesser trochanter; and canal–bone area ratios between 2–7 (CBAR2–7), 2–10 (CBAR2–10), and 7–10 cm (CBAR7–10)

Eight radiographic indices (i.e., CFI, MCI, CBR2, CBR7, CBR10, CBAR2–7, CBAR2–10, and CBAR7–10) that have demonstrated relatively good screening performance in previous studies [16–22] were measured in all patients using each patient’s digitally stored anteroposterior hip radiographs (Fig. 1). All radiographs were obtained according to our institutional preoperative imaging protocol, with the lower limbs internally rotated by 15°–20° and included a 25.4-mm-diameter metal sphere for magnification calibration. First, the femoral shaft axis was determined, and a reference line perpendicular to the femoral shaft axis was drawn through the center of the lesser trochanter and at 2 cm above and at 2, 7, and 10 cm below the lesser trochanter. Then, the CFI and MCI were measured in accordance with previous studies (Fig. 1). For CBR measurement, CBR was measured as the ratio of the canal and bone at 2, 7, and 10 cm below the lesser trochanter and defined as CBR2, CBR7, and CBR10, respectively (Fig. 1) [22]. Furthermore, three CBARs representing the areal ratios of the medullary cavity area to the total femoral cross-sectional area between 2 and 7 cm (CBAR2–7), 2–10 cm (CBAR2–10), and 7–10 cm (CBAR7–10) were calculated using the trapezoidal formula: ((top width + bottom width) × height)/2, as described in a previous study [20] (Fig. 1). The measurements were performed using Apollo View Lite (ver.4.16.8.2, https://www.vector.co.jp/soft/winnt/business/se333639.html).

### Relationship between the radiographic indices and the T-score of the proximal femur region measured by DXA

The correlation coefficients between each radiographic index and T-score were calculated. The osteoporosis screening performance of each radiographic index was calculated and compared among the indices. For indices with high diagnostic accuracy, cutoff values that could achieve a sensitivity > 90% were also investigated.

### Effect of hip diseases on osteoporosis screening using radiographic indices

An additional subgroup analysis from the primary cohort (*n* = 269) was conducted to compare patients with osteoarthritis secondary to developmental dysplasia of the hip (DDH) versus those with other diseases. We investigated whether DDH affected the diagnostic performance of each radiographic index in screening for osteoporosis; the absolute difference in cutoff values was calculated between the groups.

### Validation in an external cohort

External validation was conducted among 209 consecutive female patients who underwent primary THA at an external institution between February 2023 and December 2024. Following the same exclusion criteria used in the primary cohort (i.e., previous hip surgery, fractures, insufficient data, history of corticosteroid use, rheumatoid arthritis, or patients who received osteoporosis treatment), 61 patients were excluded, leaving 148 patients for analysis. Similar to the primary analysis, these patients had BMD measured via DXA on the surgical side.

### Assessment of intra- and interobserver reliability in measuring each radiographic index

Fifty patients were randomly selected from the primary cohort of 269 patients, and measurements were repeated by the same observer to calculate the intraobserver reliability. Furthermore, two independent observers performed additional measurements using the same protocol to calculate the interobserver reliability.

### Statistical analysis

Pearson correlation coefficients were used to assess the correlation between the radiographic indices and the T-score. Receiver operating characteristic curve analysis was performed to evaluate the osteoporosis screening performance of each radiographic index in the primary and external validation cohorts. For each index, the area under the curve (AUC), sensitivity, specificity, and the optimal cutoff value based on the Youden index were calculated. The AUC values of radiographic indices were statistically compared using DeLong’s test [24]. The interobserver and intraobserver reliability of radiographic indices were evaluated using interclass and intraclass correlation coefficient (ICC) and coefficient of variation (CV) [20, 25, 26]. For external validation, diseases were compared using the Fisher–Freeman–Halton exact test. All statistical analyses were performed using JMP Pro 17 (SAS Institute Japan, Tokyo, Japan) and MATLAB v9.10 (MathWorks, Natick, MA, USA). Statistical significance was considered at *p* < 0.05.

## Results

### Baseline patient characteristics

The study cohort had a mean age of 66.5 ± 11.0 years, height of 153.8 ± 6.4 cm, body weight of 56.3 ± 11.6 kg, and body mass index of 23.8 ± 4.6 kg/m2 (Table 1). The preoperative diagnoses included osteoarthritis secondary to DDH (*n* = 213), primary osteoarthritis (*n* = 77), rapidly destructive coxarthrosis (*n* = 14), osteonecrosis of the femoral head (*n* = 10), and psoriatic arthritis (*n* = 3). Comorbidities were identified from the electronic medical records based on categories used in previous report (Table 1) [27].

**Table 1:** Demographic characteristics and comorbidities of patients

Variable		Data
Number of patients		317
Disease (operated side)		
DDH-OA		213
Primary OA		77
RDC		14
ONFH		10
Psoriatic arthritis		3
Age (years)		66.5 ± 11.0
Height (cm)		153.8 ± 6.4
Weight (kg)		56.3 ± 11.6
BMI (kg/m^2^)		23.8 ± 4.6
T-score		− 1.9 ± 1.3
DXA to surgery date (day)		22.1 ± 10.1
Comorbidities		Number of patients
CKD (eGFR < 60 mL/min/1.73m^2^)		74
Hypogonadal states	Hypogonadism	1
Endocrine disorders	Cushing’s syndrome	1
	Diabetes mellitus	37
	Thyrotoxicosis	8
Gastrointestinal disorders	Bariatric surgery	1
	Gastrointestinal surgery	6
	Ulcerative colitis	2
	Pancreatic disease	4
Hematologic disorders	Leukemia and lymphomas	2
	Monoclonal gammopathies	1
Rheumatologic and autoimmune diseases	Ankylosing spondylitis	1
	Systemic lupus	3
	Autoimmune disease	4
Neurological and musculoskeletal risk factors	Epilepsy	1
	Parkinson’s disease	2
	Stroke	2
Other	COPD	1
	CHF	9
	Depression	9
	Sarcoidosis	1

Abbreviations: *DDH-OA*, osteoarthritis secondary to developmental dysplasia of the hip; *OA*, osteoarthritis; *RDC*, rapidly destructive coxopathy; *ONFH*, osteonecrosis of the femoral head; *BMI*, body mass index; *DXA*, dual-energy X-ray absorptiometry; *CKD*, chronic kidney disease; *eGFR*, estimated glomerular filtration rate; *COPD*, chronic obstructive pulmonary disease; *CHF*, congestive heart failure

### Osteopenia and osteoporosis prevalence and osteoporosis treatment rate

**Fig. 2:**
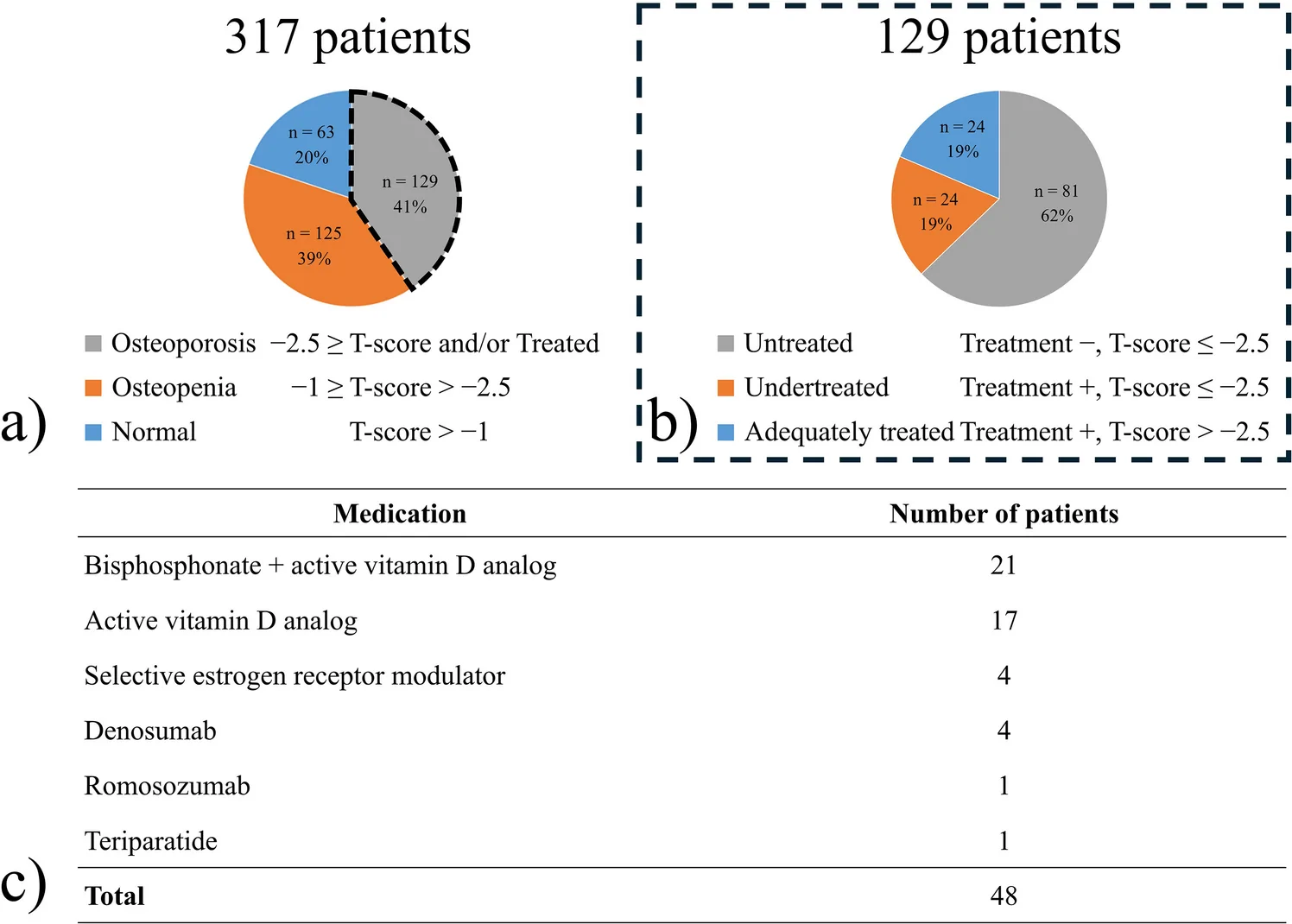
**a** Patients were categorized into osteoporosis, osteopenia, and normal based on their T-scores and treatment status. (**b**) Treatment status of 129 patients who were classified as adequately treated, undertreated, or untreated based on their osteoporosis treatment and T-scores. (**c**) Details of medications used by the 48 patients who received osteoporosis treatment

Based on hip DXA assessment of patients undergoing THA, osteopenia was found in 125 patients (39.4%), whereas osteoporosis was identified in 129 patients (40.7%) (Fig. 2a). In patients diagnosed with osteoporosis, 37.2% (48 of 129) were receiving osteoporosis treatment (Fig. 2b,c). However, 24 patients were undertreated with a T-score of ≤  − 2.5. The combined number of untreated and undertreated patients was 105 out of 129 patients, accounting for 81.4%.

### Relationship between the radiographic indices and the T-scores and osteoporosis screening performance

**Fig. 3:**
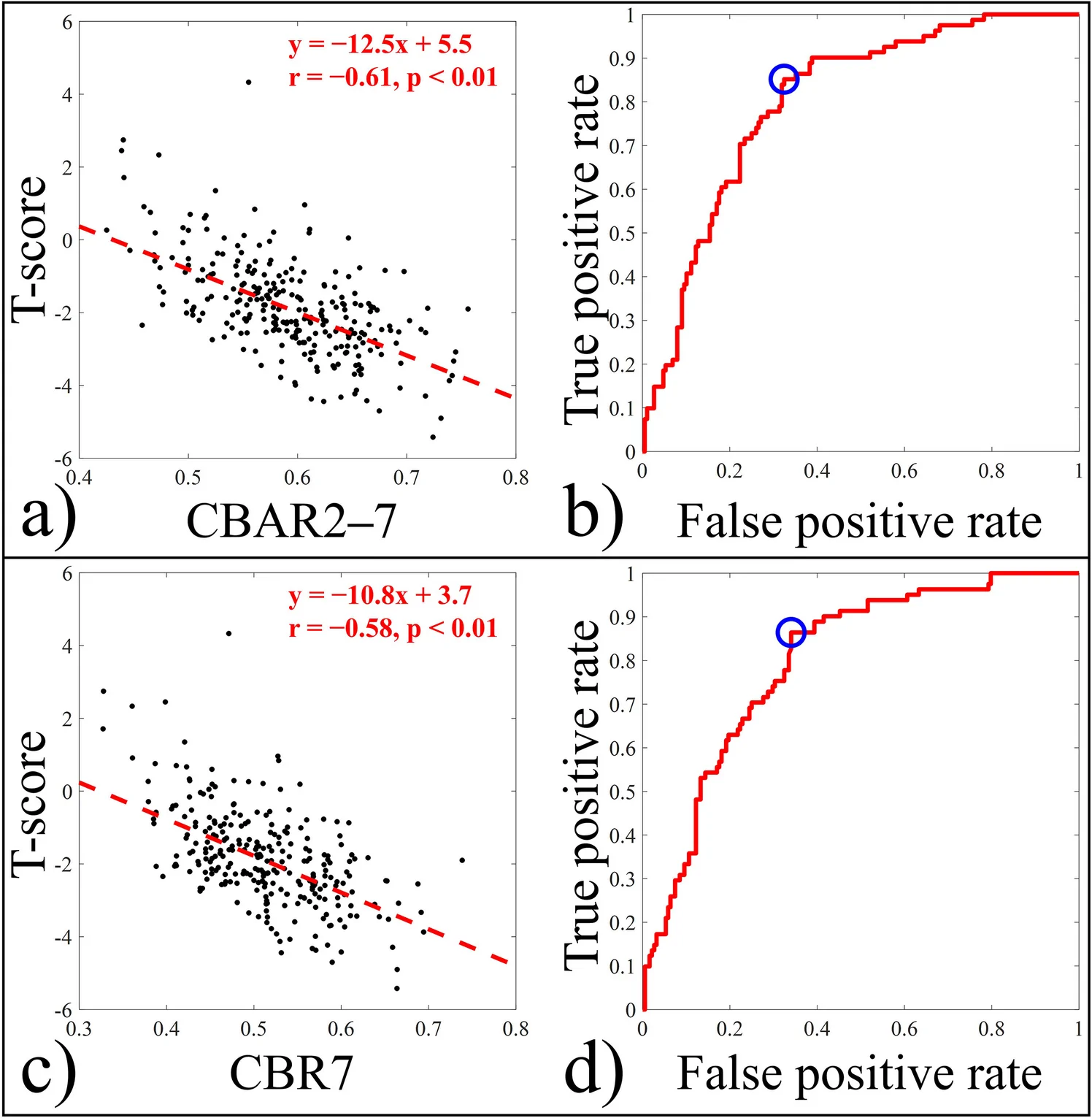
**a** Correlation between canal–bone area ratio between 2–7 cm (CBAR2–7) and T-score. (**b**) Receiver operating characteristic curve for screening osteoporosis using CBAR2–7. (**c**) Correlation between canal–bone ratio at 7 cm below the lesser trochanter (CBR7) and T-score. (**d**) Receiver operating characteristic curve for screening osteoporosis using CBR7

The correlation between the radiographic indices and the T-score ranged from *r* =  − 0.61 to 0.30, with corresponding osteoporosis screening performance showing AUC values of 0.65–0.80 (Table 2). CBAR2–7 had the highest correlation and AUC value (*r* =  − 0.61 and AUC = 0.80, respectively) (Fig. 3 a, b). Among the radiographic indices excluding CBARs, CBR7 showed the highest correlation and AUC value (Fig. 3 c, d). For CBR7, CBAR2–7, and CBAR7–10, which showed high AUC values, the cutoff values required to achieve a sensitivity of > 90% were 0.503, 0.585, and 0.485, respectively (Table 3).

**Table 2:** Correlation between radiographic indices and T-score, and osteoporosis screening performance based on receiver operating characteristic analysis

Radiographic indices	Pearson correlation (r)	Cutoff value	AUC	Sensitivity (%)	Specificity (%)
CFI	0.299	3.713	0.696	75.3	60.6
MCI	0.270	1.900	0.654	43.2	81.4
CBR2	− 0.522	0.699	0.740	60.5	78.2
CBR7	− 0.577	0.512	0.798	86.4	66.0
CBR10	− 0.551	0.491	0.766	82.7	62.2
CBAR2–7	− 0.606	0.595	0.801	85.2	67.6
CBAR2–10	− 0.599	0.574	0.785	91.4	53.7
CBAR7–10	− 0.592	0.511	0.798	84.0	71.3

Abbreviations: *AUC*, area under the curve; *CFI*, canal flare index; *MCI*, morphological cortical index; *CBR2*, canal–bone ratio at 2 cm below the lesser trochanter; *CBR7*, canal–bone ratio at 7 cm below the lesser trochanter; *CBR10*, canal–bone ratio at 10 cm below the lesser trochanter; *CBAR2–7*, canal–bone area ratio between 2–7 cm; *CBAR2–10*, canal–bone area ratio between 2–10 cm; *CBAR7–10*, canal–bone area ratio between 7–10 cm

**Table 3:** Cutoff values for ≥ 90% sensitivity and youden index–based cutoff values for CBR7, CBAR2–7, and CBAR7–10

Radiographic indices	Variable	Cutoff value achieving sensitivity > 90%	Cutoff value based on the Youden index
**CBR7**	**Cutoff value**	0.503	0.512
	**Sensitivity (**%**)**	90.1	86.4
**CBAR2–7**	**Cutoff value**	0.585	0.595
	**Sensitivity (**%**)**	90.1	85.2
**CBAR7–10**	**Cutoff value**	0.485	0.511
	**Sensitivity (**%**)**	90.1	84.0

Abbreviations: *CBR7*, canal–bone ratio at 7 cm below the lesser trochanter; *CBAR2–7*, canal–bone area ratio between 2 and 7 cm; CBAR7–10, canal–bone area ratio between 7 and 10 cm

### Comparison of AUC values across eight radiographic indices

When the AUC values were compared across indices, CBR7 and CBARs tended to have higher AUC values than other indices (Supplementary Table 1). However, no significant differences were observed among the CBR7 and three CBARs.

### Effect of hip diseases on osteoporosis screening from radiographic indices

The AUC and cutoff values were investigated between the DDH and other-disease groups. The AUC values ranged from 0.64 to 0.79 in the DDH group and 0.70–0.84 in the other-disease group. The absolute difference in cutoff values ranged from 0.007 to 0.274, and CBR7 showed the smallest difference (Supplementary Table 2).

### Validation in an external cohort

The preoperative diagnoses were comparable between the primary and external validation cohorts (*p* = 0.86). The absolute differences of cutoff values between both institutions ranged from 0.006 to 0.406 (Supplementary Table 3).

### Interobserver and intraobserver reliability in measuring each radiographic index

For the interobserver reliability, the CV ranged from 3.46% to 6.31%, with MCI showing the highest CV. Meanwhile, the ICC ranged from 0.560 to 0.882, with CBAR7–10 showing the highest interobserver reliability (Supplementary Table 4). For the intraobserver reliability, the CV ranged from 1.39% to 3.25%, with MCI showing the highest CV. Meanwhile, the ICC ranged from 0.827 to 0.952, with CBAR7–10 showing the highest intraobserver reliability (Supplementary Table 4).

## Discussion

In this study, we found that female patients scheduled to undergo THA had a high prevalence of osteoporosis (40.7%) and a low treatment rate (37.2%). This finding highlights the importance of osteoporosis screening for these patients. When osteoporosis was screened using eight hip radiographic indices on anteroposterior hip radiograph, CBR7 and CBAR2–7, CBAR2–10, and CBAR7–10 demonstrated the highest screening accuracy with no difference among them. Considering that CBR7 is a simple index, it is recommended to be used by surgeons for osteoporosis screening.

### Osteopenia and osteoporosis prevalence and osteoporosis treatment rate

In this cohort of preoperative female patients undergoing THA, osteopenia was identified in 125 out of 317 patients (39.4%), which was comparable to previous reports ranging from 37.9% to 46.2% [7–9]. By contrast, osteoporosis was identified in 129 out of 317 patients (40.7%), and only 48 of these patients (37.2%) received treatment. This prevalence rate was higher than previously reported rates (8.7%–25.0%, Supplementary Table 5). Although the patient background in our study (only females with relatively low body weight) likely affected the results, it is important to highlight the high proportion of osteoporosis in female patients undergoing THA. With regard to the treatment rate, the combined proportion of undertreated (patients who were receiving osteoporosis treatment but had a T-score of ≤  − 2.5) and untreated patients reached 81.4%. In this study, osteoporosis was defined as a T-score ≤  − 2.5 at the hip undergoing THA because our objective was to evaluate BMD on the surgical side. However, the clinical diagnosis of osteoporosis is generally based on the lowest T-score across multiple skeletal sites, including the lumbar spine and hip. Thus, the prevalence of osteoporosis was likely underestimated in this study. Nevertheless, even with our limited definition, a considerable number of patients scheduled to undergo THA were found to have untreated osteoporosis. These findings support the clinical relevance of this study and highlight the importance of preoperative screening for osteoporosis.

### Relationship between the radiographic indices and the T-scores and osteoporosis screening performance

The CTI and CBR provide similar information when measured at the same femoral level [28, 29]. Therefore, to avoid overlaps, this study evaluated CBR across different femoral levels. CBR represents the ratio of the medullary canal width to the total femoral width, whereas CBAR represents the ratio of the medullary canal area to the total femoral area. In previous studies, CFI, MCI, CBR, and CBAR demonstrated correlations with T-scores ranging from (|r|= 0.36 to 0.77 [19–22], which were consistent with those in the present study (|r|= 0.27–0.61).

When the radiographic indices were used to screen for osteoporosis, CFI and MCI showed lower screening performance, whereas CBR7, CBR10, and CBARs showed higher AUC values. These findings are consistent with previous studies that reported AUC values of 0.544–0.800 for CFI and MCI [19–22] and 0.732–0.821 for CBR and CBAR. Although the cohort in the present study included only females and differed from those of Watanabe et al. and Rohe et al., it demonstrated comparable correlations and screening performance, thereby supporting the clinical relevance of comparing osteoporosis screening performance across different femoral levels [7, 22]. For CBR and CBAR, lowering the threshold can increase sensitivity and reduce missed cases (Table 3), but it also increases the number of unnecessary DXA assessments done. Therefore, establishing a cutoff value that strikes a balance between sensitivity and specificity is necessary to identify patients who need further evaluation with DXA.

When compared across radiographic indices, the osteoporosis screening performance of CBR7 and CBARs was higher than that of CFI, MCI, CBR2, and CBR10. No significant differences in AUC values were observed among the CBR7 and three CBSRs, indicating that these four indices are effective and can be used in the clinic to screen for osteoporosis. In previous studies, reduced femoral BMD was associated with cortical thinning and medullary canal expansion among Japanese women [30, 31]. As CBR7 is based on the ratio of the medullary canal width to the total femoral width, it may reflect such structural changes related to bone loss. Additionally, femoral BMD is influenced by both cortical and trabecular bone; these components may have similar contributions at the femoral shaft.

CBARs require measurements at multiple points, but routine hip radiographs do not always capture the region 10 cm below the lesser trochanter. In contrast, CBR7 is simpler to measure and can be applied to a wider range of radiographs. Although CBR can be measured at several levels along the femoral shaft, CBR7 demonstrated consistently high interobserver and intraobserver ICCs. This consistency in measurements may have also contributed to its favorable screening performance. Therefore, the choice of index should consider not only screening performance but also measurement burden and image availability.

### Effect of hip diseases on osteoporosis screening using radiographic indices

The AUC values tended to be lower in the DDH group than in the other-disease group. Since the two groups represented different cohorts, statistical comparisons of the AUC values using DeLong’s test could not be performed. Nevertheless, the overall pattern was consistent between the subgroup analysis and the primary cohort. CFI and MCI showed low AUCs, whereas CBRs and CBARs showed high AUCs. The absolute differences in cutoff values between both groups were < 0.05 for all CBRs and CBARs, and CBR7 had the smallest difference at 0.007. Thus, the influence of DDH on the diagnostic performance of these radiographic indices for osteoporosis may be limited to CBRs and CBARs.

### Validation in an external cohort

During external validation, the absolute differences in cutoff values between the primary cohort and external institution were less than 0.05 for all CBRs and CBARs. Specifically, the differences in cutoff values for the CBRs were small (< 0.009). Thus, the radiographic indices evaluated in the main study maintained acceptable diagnostic accuracy in the external cohort.

### Interobserver and intraobserver reliability in measuring each radiographic index

All indices except for CBR2 and MCI demonstrated interobserver ICC values > 0.75. Meanwhile, all indices showed intraobserver ICC values > 0.80. These results are consistent with those of previous studies that reported interobserver and intraobserver ICC values of 0.70–0.97 for CBR and CFI [19, 22, 32]. Importantly, the ICC values of CBR7 and CBARs remained high, further supporting the findings of this study.

### Application of our results for clinical practice

This study has two important clinical implications. First, a considerable number of female patients scheduled to undergo THA also had osteoporosis that was not adequately treated, highlighting the need for osteoporosis screening among this patient population. Second, we investigated 8 radiographic indices and directly compared them within the same cohort, which can be used to screen for patients who require further investigation with DXA with a sensitivity of 80%–90%.

CBARs involve measurements at multiple points, which require extra time and effort. Meanwhile, CBR7 and CBR10 demonstrated comparable reliability to CBARs while being simpler to measure. However, it must be noted that the region 10 cm below the lesser trochanter is not always captured in routine hip radiographs. Additionally, the CBR7 may have broader clinical applicability, as evidence by its smaller CV and larger ICC compared to the CBR10. As the CBR7 had a cutoff value of 0.51, surgeons can effectively screen for osteoporosis by simply assessing whether the cancellous portion of the bone is larger than half of the total width at 7 cm below the lesser trochanter.

Recent advancements in artificial intelligence have enabled rapid, automated osteoporosis screening using hip radiographs [33–35]. However, these are not universally available in clinical practice. Many artificial intelligence models are also difficult to interpret, and the radiographic features used for decision-making remain unclear. Our findings may help clarify these radiographic features by demonstrating the relationship of radiographic indices with femoral BMD.

### Limitations

This study has some limitations. First, the single-center retrospective design of this study may have introduced selection bias. To address this limitation, we conducted an external validation in an independent cohort from another institution. This revealed similar diagnostic performance and cutoff values, supporting the reproducibility of the primary findings. Second, we evaluated each radiographic index alone in a cohort limited to Japanese female patients undergoing THA. Although osteoporosis is predominant among females [36], further validation is needed among men, healthy individuals, other clinical populations, and different ethnic groups to confirm the generalizability of these findings, especially because parameters such as femoral morphology can differ based on sex and ethnicity. Third, our assessment was solely based on anteroposterior hip radiographs. Incorporating several variables such as multiple radiographic views (e.g., lateral view), clinical information (e.g., age and weight), and screening tools (e.g., FRAX) may further improve the accuracy of screening. However, this approach deviates from the primary aim of providing a simple screening tool [37, 38]. Furthermore, the Dorr classification using lateral radiographs has shown only moderate interobserver agreement and may be affected by patient positioning. Thus, its application should be considered carefully in clinical practice [39].

## Conclusions

In female patients scheduled to undergo THA, the osteoporosis prevalence and treatment rates were 40.7% and 37.2%, respectively. CBR7 with a cutoff value of 0.51 demonstrated comparable osteoporosis screening performance to that of CBARs, suggesting its potential utility as a simple screening tool for osteoporosis.

## Supplementary Information

Below is the link to the electronic supplementary material.Supplementary file1 (DOCX 34.2 KB)Supplementary file2 (DOCX 34.9 KB)Supplementary file3 (DOCX 35.3 KB)Supplementary file4 (DOCX 34.8 KB)Supplementary file5 (DOCX 34.3 KB)

## Data Availability

The datasets generated and/or analyzed during the current study are not publicly available due to restrictions related to patient privacy and institutional regulations but are available from the corresponding author on reasonable request and with permission from Osaka University Hospital and Japan Community Health Care Organization Osaka Hospital.
