# Supplementary material for: Hip anteroposterior radiographic indices for effective osteoporosis screening in female patients undergoing total hip arthroplasty
Source: Arch Osteoporos. 2026 Aug 28;21(1):132. doi: 10.1007/s11657-026-01760-3 (PMC13525003; doi:10.1007/s11657-026-01760-3)
Supplement: Supplementary file 1 [file 11657_2026_1760_MOESM1_ESM.docx]

**Supplementary table 1.** P-values of pairwise comparisons of area under the curves among radiographic indices

| X-ray indices | MCI | CBR2 | CBR7 | CBR10 | CBAR2–7 | CBAR2–10 | CBAR7–10 |
| --- | --- | --- | --- | --- | --- | --- | --- |
| CFI | 0.140 | 0.284 | <0.001 | 0.002 | 0.002 | 0.004 | <0.001 |
| MCI | – | 0.064 | <0.001 | <0.001 | <0.001 | <0.001 | <0.001 |
| CBR2 | – | – | 0.063 | 0.448 | <0.001 | 0.008 | 0.065 |
| CBR7 | – | – | – | 0.114 | 0.851 | 0.540 | 0.944 |
| CBR10 | – | – | – | – | 0.160 | 0.337 | 0.003 |
| CBAR2–7 | – | – | – | – | – | 0.163 | 0.901 |
| CBAR2–10 | – | – | – | – | – | – | 0.451 |

Abbreviations: CFI, canal flare index; MCI, morphological cortical index; CBR2, canal–bone ratio at 2 cm below the lesser trochanter; CBR7, canal–bone ratio at 7 cm below the lesser trochanter; CBR10, canal–bone ratio at 10 cm below the lesser trochanter; CBAR2–7, canal–bone area ratio between 2–7 cm; CBAR2–10, canal–bone area ratio between 2–10 cm; CBAR7–10, canal–bone area ratio between 7–10 cm
