# Supplementary material for: Hip anteroposterior radiographic indices for effective osteoporosis screening in female patients undergoing total hip arthroplasty
Source: Arch Osteoporos. 2026 Aug 28;21(1):132. doi: 10.1007/s11657-026-01760-3 (PMC13525003; doi:10.1007/s11657-026-01760-3)
Supplement: Supplementary file 2 [file 11657_2026_1760_MOESM2_ESM.docx]

**Supplementary table 2**. Influence of developmental dysplasia of the hip on the accuracy of radiographic indices for osteoporosis screening

| **Radiographic indices** | **DDH group** | | **Other-disease group** | | **Absolute difference in cutoff values between groups** |
| --- | --- | --- | --- | --- | --- |
|  | **AUC** | **Cutoff value** | **AUC** | **Cutoff value** |  |
| CFI | 0.677 | 3.900 | 0.739 | 3.713 | 0.187 |
| MCI | 0.635 | 2.212 | 0.699 | 1.938 | 0.274 |
| CBR2 | 0.717 | 0.702 | 0.770 | 0.654 | 0.048 |
| CBR7 | 0.787 | 0.512 | 0.811 | 0.505 | 0.007 |
| CBR10 | 0.745 | 0.470 | 0.812 | 0.487 | 0.017 |
| CBAR2–7 | 0.779 | 0.585 | 0.821 | 0.602 | 0.017 |
| CBAR2–10 | 0.762 | 0.574 | 0.822 | 0.608 | 0.034 |
| CBAR7–10 | 0.781 | 0.519 | 0.836 | 0.508 | 0.011 |

Abbreviations: DDH-OA, osteoarthritis secondary to developmental dysplasia of the hip; AUC, area under the curve; CFI, canal flare index; MCI, morphological cortical index; CBR2, canal–bone ratio at 2 cm below the lesser trochanter; CBR7, canal–bone ratio at 7 cm below the lesser trochanter; CBR10, canal–bone ratio at 10 cm below the lesser trochanter; CBAR2–7, canal–bone area ratio between 2–7 cm; CBAR2–10, canal–bone area ratio between 2–10 cm; CBAR7–10, canal–bone area ratio between 7–10 cm
