# Supplementary material for: Hip anteroposterior radiographic indices for effective osteoporosis screening in female patients undergoing total hip arthroplasty
Source: Arch Osteoporos. 2026 Aug 28;21(1):132. doi: 10.1007/s11657-026-01760-3 (PMC13525003; doi:10.1007/s11657-026-01760-3)
Supplement: Supplementary file 3 [file 11657_2026_1760_MOESM3_ESM.docx]

**Supplementary table 3**. External validation of cutoff values for radiographic indices

| **Variable** | | **Data** | | ***p*-value** |
| --- | --- | --- | --- | --- |
|  | | **Our institution** | **External institution** |  |
| Number of patients (n) | | 269 | 148 | - |
| Disease (n) | |  |  |  |
|  | DDH-OA | 177 | 95 | 0.86 |
|  | Primary OA | 70 | 42 |  |
|  | RDC | 12 | 8 |  |
|  | ONFH | 7 | 2 |  |
|  | Other | 3 | 1 |  |
| **Radiographic indices** | | **Cutoff vales** | | **Absolute difference of cutoff values** |
|  |  | **Our institution** | **External institution** |  |
| CFI | | 3.713 | 3.307 | 0.406 |
| MCI | | 1.900 | 1.918 | 0.018 |
| CBR2 | | 0.699 | 0.705 | 0.006 |
| CBR7 | | 0.512 | 0.504 | 0.008 |
| CBR10 | | 0.491 | 0.483 | 0.008 |
| CBAR2–7 | | 0.595 | 0.626 | 0.031 |
| CBAR2–10 | | 0.574 | 0.621 | 0.047 |
| CBAR7–10 | | 0.511 | 0.490 | 0.021 |

Abbreviations: DDH-OA, osteoarthritis secondary to developmental dysplasia of the hip; OA, osteoarthritis; RDC, rapidly destructive coxopathy; ONFH, osteonecrosis of the femoral head; CFI, canal flare index; MCI, morphological cortical index; CBR2, canal–bone ratio at 2 cm below the lesser trochanter; CBR7, canal–bone ratio at 7 cm below the lesser trochanter; CBR10, canal–bone ratio at 10 cm below the lesser trochanter; CBAR2–7, canal–bone area ratio between 2–7 cm; CBAR2–10, canal–bone area ratio between 2–10 cm; CBAR7–10, canal–bone area ratio between 7–10 cm
