# Supplementary material for: Hip anteroposterior radiographic indices for effective osteoporosis screening in female patients undergoing total hip arthroplasty
Source: Arch Osteoporos. 2026 Aug 28;21(1):132. doi: 10.1007/s11657-026-01760-3 (PMC13525003; doi:10.1007/s11657-026-01760-3)
Supplement: Supplementary file 4 [file 11657_2026_1760_MOESM4_ESM.docx]

**Supplementary table 4.** Inter- and intraobserver reliability of radiographic indices

| **Interobserver reliability** | | |
| --- | --- | --- |
| **Radiographic indices** | **CV (**%**)** | **Interclass correlation coefficient** |
| CFI | 5.37 | 0.799 |
| MCI | 6.31 | 0.671 |
| CBR2 | 5.47 | 0.560 |
| CBR7 | 3.99 | 0.854 |
| CBR10 | 4.50 | 0.820 |
| CBAR2–7 | 3.95 | 0.783 |
| CBAR2–10 | 4.04 | 0.763 |
| CBAR7–10 | 3.46 | 0.882 |
| **Intraobserver reliability** | | |
| **Radiographic indices** | **CV (**%**)** | **Intraclass correlation coefficient** |
| CFI | 2.93 | 0.896 |
| MCI | 3.25 | 0.827 |
| CBR2 | 2.42 | 0.865 |
| CBR7 | 2.01 | 0.932 |
| CBR10 | 2.54 | 0.887 |
| CBAR2–7 | 1.66 | 0.946 |
| CBAR2–10 | 1.39 | 0.939 |
| CBAR7–10 | 1.57 | 0.952 |

Abbreviations: CV, coefficient of variation; CFI, canal flare index; MCI, morphological cortical index; CBR2, canal–bone ratio at 2 cm below the lesser trochanter; CBR7, canal–bone ratio at 7 cm below the lesser trochanter; CBR10, canal–bone ratio at 10 cm below the lesser trochanter; CBAR2–7, canal–bone area ratio between 2–7 cm; CBAR2–10, canal–bone area ratio between 2–10 cm; CBAR7–10, canal–bone area ratio between 7–10 cm
