# Supplementary material for: Hip anteroposterior radiographic indices for effective osteoporosis screening in female patients undergoing total hip arthroplasty
Source: Arch Osteoporos. 2026 Aug 28;21(1):132. doi: 10.1007/s11657-026-01760-3 (PMC13525003; doi:10.1007/s11657-026-01760-3)
Supplement: Supplementary file 5 [file 11657_2026_1760_MOESM5_ESM.docx]

| **Authors** | **Number of patients (n)** | **Male/female (n)** | **Age (years)** | **Region of diagnosis** | **Osteopenia prevalence (**%**)** | **Osteoporosis prevalence (**%**)** |
| --- | --- | --- | --- | --- | --- | --- |
| Delsman et al. [7] | 268 | 90/178 | >70 | Hip or spine | 41.0 | 18.3 |
| Watanabe et al. [8] | 398 | 68/330 | 65 ± 12 | Hip | 46.2 | 8.7 |
| Domingues et al. [9] | 29 | 13/16 | 62 ± 15 | Hip or spine | 37.9 | 20.7 |
| Glowacki et al. [10] | 68 | 0/68 | 46–89 | Hip or spine | - | 25.0 |
| Present study | 317 | 0/317 | 67 ± 11 | Hip | 39.4 | 40.7 |

**Supplementary table 5.** Summary of osteopenia and osteoporosis prevalence reported in previous studies for patients undergoing total hip arthroplasty
